# The end of the era of generosity? Global health amid economic crisis

**DOI:** 10.1186/1747-5341-4-1

**Published:** 2009-01-09

**Authors:** Kammerle Schneider, Laurie Garrett

**Affiliations:** 1 Global Health Program, Council on Foreign Relations, New York, NY, USA

## Abstract

In the past decade donor commitments to health have increased by 200 percent. Correspondingly, there has been a swell of new players in the global health landscape. The unprecedented, global response to a single disease, HIV/AIDS, has been responsible for a substantial portion of this boon. Numerous health success have followed this windfall of funding and attention, yet the food, fuel, and economic crises of 2008 have shown the vulnerabilities of health and development initiatives focused on short term wins and reliant on a constant flow of foreign funding. For too long, the international community has responded to global health and development challenges with emergency solutions that often reflect the donor's priorities, values, and political leanings, rather than funding durable health systems that can withstand crises. Progress towards achieving the Millennium Development Goals has stalled in many countries. Disease specific initiatives have weakened health systems and limited efforts to improve maternal and child health. As we enter this era of scarce resources, there is a need to return to the foundations of the Alma Ata Declaration signed thirty years ago with the goal of providing universal access to primary healthcare. The global health community must now objectively evaluate how we can most effectively respond to the crises of 2008 and take advantage of this moment of extraordinary attention for global health and translate it into long term, sustainable health improvements for all.

## Introduction

Over the past eight years global health has taken center stage in an era of historic generosity as the wealthy world has committed substantial resources to tackle poverty and disease in developing countries. Between 2000 and 2006, estimated donor commitments for global health increased by 200 percent – from $15 billion to $45 billion [1]. Correspondingly, there has been a massive swell in the number of nonprofit organizations (NGOs), faith based groups, and private actors contributing to this boon.

Remarkable achievements have followed this windfall of funding and attention, including numbers of lives saved, children vaccinated, people placed on HIV/AIDS medication, institutional improvements, and rising commitment by developing countries themselves, to the public goods needs of their people. But the economic, food, and fuel crises of 2008 threaten to erase these achievements, pushing those peoples of the world that saw hope on their horizons back into dire poverty, disease, and despair. Will rising food costs, economic uncertainty, and an increased focus on problems within their own borders erode the wealthy world's commitment to poverty eradication and global health as we enter an era of scarce resources?

## Past is prelude

Thirty years ago, in the midst of the Cold War, representatives from 134 World Health Organization (WHO) member states gathered in the former Soviet Republic city of Alma-Ata. East and west, north and south convened to discuss how to provide essential public health goods and access to healthcare for the world's poorest. More historic than the gathering of communist and capitalist nations, was the recognition by all parties of health as a key determinant of development rather than an outcome of medical interventions. On September 12, 1978 the Alma-Ata Declaration was born, stating that primary health care "based on practical, scientifically sound, and socially acceptable methods and technology made universally acceptable through people's full participation," [2] was key to meeting the goal of providing health care for all by the year 2000. In the past three decades progress has been made. A baby born in Alma-Ata in 1978 had a 7.3 percent risk of dying before his or her fifth birthday. The risk for a baby born today, in what is now Almaty, Kazakhstan, is now only 2.9 percent and this reduction mirrors the average worldwide reduction in child mortality over the last thirty years [3]. Yet today, the landscape of global health is drastically different from that of three decades ago. The effects of globalization, spread of infectious diseases, rapid urbanization, and increasing disparities between rich and poor have, by necessity, shifted the world's focus from meeting the goal of access to universal primary health care to finding emergency, stopgap solutions to ease the suffering caused by high mortality crises, such as HIV/AIDS and humanitarian disasters.

The emergence of HIV/AIDS fundamentally transformed the way in which the world engaged global health. It shook world leaders out of a long period of a smug belief that microbes would be conquered as a corollary of rising economic growth. It also awoke the average citizen to the gross disparities in access to health that exist between rich and poor countries, mobilizing remarkable numbers of wealthy world citizens to take action on behalf of people living both great cultural and physical distances from themselves. The political zeal and advocacy efforts generated by the AIDS pandemic pushed health to the top of the international development agenda. The fight against HIV/AIDS rallied tremendous political and financial support for global health, while at the same time, moving investments in health from infrastructure: clinics, roads, clean water, sanitation, medical supplies, and the training and management of skilled medical personnel, to funding disease specific initiatives with emergency, short term targets, and often unsustainable results.

Building on the heightened attention to global health issues, during the 1990s, the international community developed the Millennium Development Goals (MDGs), a set of ambitious targets to reach by 2015 with the overall goal of reducing global poverty and improving the health and welfare of the world's poor. Three of the eight MDGs relate specifically to health issues and others address the interconnected nature of health and development through sanitation, education, and poverty alleviation. As governments and private institutions began to confront the HIV/AIDS pandemic and strive to meet the MDGs, there was a growing sentiment that the traditional system of bilateral agencies and international organizations serving as the primary implementers of global health policies and programming was insufficient.

Over the past decade, there has been a massive increase in new global health players. Private foundations, such as the Bill and Melinda Gates Foundation, innovative global funds, such as the GAVI Alliance and the Global Fund to Fight AIDS, Tuberculosis and Malaria (GFATM), and engaged corporate actors have transformed the landscape of global health with their access to substantive funding streams and ability to respond more rapidly to the perceived needs on the ground. In addition to these multilateral initiatives, the United States, under the Bush Administration, made an unprecedented bilateral commitment to HIV/AIDS in 2003 under the President's Emergency Plan For AIDS Relief (PEPFAR) and to malaria as part of the President's Malaria Initiative (PMI). The excitement generated by new global health players and monies has mobilized the citizenry of wealthy countries – whether buying a red iPod as part of the RED campaign to support HIV/AIDS efforts, or wearing a white bracelet to raise awareness of issues of global poverty in the ONE campaign – the average rich world citizen is engaged in global health like never before.

## Building on success

Since 2000, great achievements have been made. By October 2008, the GFATM had dispersed $6.4 billion worth of grants for country-designed programs in a mechanism that is both without precedent, and empirically successful in achieving its targets some 80 percent of the time [4]. PEPFAR had, by March 31, 2008, started 1.73 million people on antiretroviral treatment for HIV infection, and provided antiretroviral prophylaxis for more than one million pregnant women to prevent infant in utero infection [5]. The combined donor, GFATM, and United Nations (UN) efforts to tackle malaria had, by the end of 2008, pushed down deaths due to malaria by fifty percent in key African and Asian countries, in large part due to dispersal of pesticide-treated mosquito nets and insecticide spraying campaigns [6].

2008 has been a historic year for global health. In June, the U.S. Congress passed the PEPFAR reauthorization act with nearly unanimous support, raising the U.S. commitment to treating and preventing HIV/AIDS and Malaria, and Tuberculosis infections in fifteen target countries to an astounding $48 billion over the course of the next five years [7]. In September, the UN, in partnership with a variety of governments and NGOs, launched the Global Malaria Action Plan – aimed at eradicating Malaria worldwide by 2015 [8]. The effort has received a $3 billion dollar boost from a dozen organizations, led by the World Bank and the Bill and Melinda Gates Foundation [9].

Yet, despite large investments, audacious goals, and widespread attention and support for global health, 2008 has also been a year that has brought into question the sustainability and durability of many of these efforts. The economic, food, and fuel crises of 2008 have shown the vulnerabilities of health systems reliant on a constant flow of foreign funding. For too long, the international community has responded to global health and development challenges with emergency, short term solutions that often reflect the donor's priorities, values and political leanings, rather than funding durable health systems that can withstand crises.

In October 2008, the World Bank President, Robert Zoellick, warned, "While people in the developed world are focused on the financial crisis, many forget that a human crisis is rapidly unfolding in developing countries. It is pushing poor people to the brink of survival," where the number of malnourished people globally will grow by forty-four million, to 967 million in 2008, as several countries experienced double-digit food inflation [10].

The food crisis has shown how unprepared health authorities often are to changes in the broader environment. In the past year, the cost of wheat has risen by 130 percent, rice by 120 percent, with corn and soy prices not far behind. As a result, millions of people are starving and at least 100 million more people will be pushed further into poverty [11]. The International Fund for Agricultural has estimated that the number of food-insecure people in the world will rise by sixteen million for every percentage increase in the prices of staple goods [12]. We have only begun to witness the impact of hedge fund trading in New York on the lives of some two billion people living in poverty. Instead of investing in long term agricultural development schemes, the majority of donor funding over the past decade has focused on providing emergency food aid to countries on the brink of widespread famine. We have jumped from one emergency band-aid solution to the next, instead of focusing on the structural causes of food insecurity.

2008 also marks the midway point for achievement of the MDGs [13]. In September, the Office of the UN Secretary General concluded that both funding and program development were falling far short of those needed to reach the 2015 MDGs, and at least six of the eight targets were on course to fail. MDG 5 – maternal survival – has not shown significant improvement and no region is on track to achieve the goal at current rates [13]. The target of MDG 1 – to reduce the proportion of people who suffer from extreme poverty and hunger – is in reverse. Well before the impact of the financial meltdown was felt, donor support had declined. Aid dropped 8.4 percent in 2007, after a 4.7 percent drop in 2006. The Group of 8 industrialized nations pledged in 2005 to donate more than $25 billion to Africa by 2010, but just $4 billion has actually been delivered [14].

International institutions and governments heavily reliant on steady inflow of foreign donor funding are now frantically trying to resolve how to continue the operations of their health programs, as wealthy nations are paying hundreds of billions to rescue the world's financial industry. Undoubtedly, the economic crisis will crimp humanitarian aid, and international efforts to fight disease and alleviate poverty. Philanthropic giving from governments, foundations, and corporations is expected to sharply decline as the world tightens its belt and heads into a global recession. "It is not clear what the current financial crisis will mean for low income and emerging economies, but many predictions are highly pessimistic. Margaret Chan, Director General of the WHO, warned in a statement. "In the face of a global recession, fiscal pressures in affluent countries may prompt cuts to official development assistance" [15].

As evidenced over the past thirty years, increased commitments to global health do not automatically equate to sustainable changes in health in individual countries, especially among the poorest of poor. Although health indicators have improved among some groups, we have seen increases in gaps in health outcomes among women, children, and marginalized populations, across regions and within countries. In poor rural areas of western China, the maternal mortality ratio is four times that of urban areas and double that of rural areas in eastern China [16]. Despite the great efforts of many organizations, life expectancy has barely budged in these populations.

Today, the global life expectancy gap is the widest in human history, with a disparity of nearly five decades. Each day around 28,000 children under five die from largely preventable causes and every minute of every day a woman dies of pregnancy-related complications. Recent UN data on maternal mortality show that a woman living in Afghanistan or Sierra Leone has a one in eight chance of dying in pregnancy or childbirth. This compares with a one in 4,800 risk for a woman in the United States, and a more than one in 17,400 risk for a mother in Sweden [17]. This logarithmic differential in maternal survival represents the most striking, even egregious, health disparity in the twenty-first century world.

## The special challenge of HIV

Increased focus on the urgent management of specific diseases has weakened the ability of health systems to respond to crises. To respond to the AIDS epidemic, the share of global health aid devoted to HIV/AIDS more than doubled between 2000 and 2004 – reflecting the global response to an important need, yet, the share devoted to primary care dropped by almost half during the same time period [18]. Enhancing one program, at the apparent cost to another, merely shifted the face of catastrophe from one health paradigm, to another.

With increased funding, the world has made progress towards the goal of universal access to HIV/AIDS treatment. The number of people on antiretroviral therapy (ARVs) has increased from two per cent to twenty-eight per cent in the last four years [19]. For international donors, making a commitment to provide treatment comes with great responsibility and an ever-increasing price tag. As the number of people infected grows, the number of people that require second line, more expensive, drugs swells. But treatment alone will not end the AIDS pandemic. For every HIV+ individual that went on ARVs in 2006, six more people contracted the virus [20,21].

The current focus on ARVs risks creating a medicine-dominated response to HIV/AIDS, and diverting attention and funds away from the more fundamental political, social, and economic determinants of poverty and the spread of infectious disease. Many current initiatives are trying to build dams – pharmaceutical dams to hold back the pandemic – but behind those dams the number of newly infected keeps rising, threatening to overflow and drown these efforts.

In many of the countries hardest hit by the pandemic, a large portion of their funding for AIDS medications come from outside donors. For example, in Mozambique, 98 percent of all funding for the country's HIV/AIDS programs comes from outside donors: 78 percent of it is from the U.S. PEPFAR program. Similarly, Uganda is 95 percent dependent on external donors for financing of its HIV/AIDS programs: 73 percent of outside support is from the U.S. PEPFAR program [22]. In both of these cases the nation's extraordinary dependence on external support begs questions about the efforts' sustainability, and country ownership and control. Were the U.S. to suddenly cease underwriting these programs, AIDS patients would die by the thousands for lack of life-extending treatment. As we enter an economic downturn, the sustainability of emergency initiatives, such as PEPFAR, that are 100 percent dependent on a never ending supply of donor dollars, are called into question [23].

## Moral hazard amid complexity

Instead of making things simpler and more efficient on the ground, in many cases, the rapid increase in funding and number of global health players has made the mechanisms for delivering aid even more complex. At the developing country level, where these activities are targeted, hundreds of foreign entities are competing for the attention of local governments, civil society interest, and the desperately short supply of trained healthcare workers. Ministers of Health in recipient countries say that their days are over burdened by long lines of NGOs and bilateral program contractors, each demanding their attention. In Mozambique, for example, there are fifty distinct donors funding health and development programming in the country. Of these, nineteen are providing foreign aid directly to the government through budgetary support while the majority provides aid through their own individualized mechanisms or agreements which each require their own monitoring and reporting requirements from recipients [24].

Further exacerbating the difficulties of responding to the health needs of the world's poorest is the current state of health systems and capacity in the many developing countries. Decades of neglect, coupled with austerity programs imposed by the International Monetary Fund in the 1980s and 1990s, have rendered hospitals, clinics, laboratories and health care workers dangerously deficient. According to the WHO's World Health Report 2006, there is a shortage of more than four million health care workers in 57 developing countries [25]. Compounding the problem, local healthcare workers often grow so exasperated and demoralized by their dysfunctional health systems that they apply for higher paying jobs abroad, thus accelerating a 'brain drain' at home. One quarter of physicians and one in 20 nurses trained in Africa currently work in the 30 industrialized countries in the Organization for Economic Cooperation and Development (OECD) [26]. There is also an internal brain drain within countries as healthcare workers leave public hospitals and health centers lured by more lucrative jobs in clinics run by foreign NGOs, bilateral donors, and faith-based organizations. In Ethiopia, contract staff hired to help implement disease specific programs earned salaries three times greater than regular government health employees [27] and in Malawi, one hospital reported that 88 of its nurses left within an eighteen month period to take better paying jobs in NGOs programs [28].

## A moral path forward

Progress towards achieving the MDGs has slowed in many countries. Disease specific initiatives have weakened health systems and limited efforts to improve maternal and child health and our ability to respond to new health and development crises. The threats posed by newly emerging infectious diseases, climate change, urbanization, and the rise of chronic diseases threatens to erase many of the gains we have achieved. Thirty years on, the concept of providing primary health care for all offers a possible roadmap to attain the MDGs by 2015 and create sustainable, long term investments in health. It is heartening to see that global health leaders have recognized the urgent need to create greater coherence among health initiatives and organizations, and focus funding and attention on basic health system investments to save millions of people every year that now perish needlessly from preventable diseases and find new tools to save *still more *lives.

Given the scale of the world's healthcare workers deficit, no progress can be made in the creation of universal primary care systems if models continue to be doctor-based. Even if the world committed today to the most massive medical training exercise in history, the deficit would not be overcome for more than two generations. Only a substantial commitment to building genuinely viable health infrastructures centered on community based workforces, coupled with local profit incentive systems, and global scale supply and inventory management can create primary health systems that can prevent hundreds of millions of deaths due to childbirth complications, pediatric diarrheal diseases, infectious diseases, and the newly emerging chronic diseases of diabetes, heart disease, and cancer.

The crises of 2008 have brought together committed government officials, UN agency leaders, NGOs, faith-based groups, and corporate actors to collectively think about new ways to break out of patterns of charitable giving and move towards real sustainable investments in health utilizing the wealth of resources and technical expertise available both on the ground and within international agencies. A number of promising initiatives, commitments, and programs are beginning to emerge in an effort to improve global health funding efficacy through longer term commitments, more coordinated accountability measures, and inner-agency collaboration mechanisms.

Within the UN system, efforts are underway to improve relations between health-focused UN agencies, and large global initiatives, including, the GAVI Alliance, the Global Fund to Fight AIDS, Tuberculosis and Malaria, and the Bill & Melinda Gates Foundation. Calling itself the H-8 (health-8: WHO, UNICEF, UNAIDS, UNFPA, World Bank, GFATM, GAVI, and Gates), this alliance has set its top management tiers to the task of talking to one another on a regular basis to clarify the core responsibilities of each agency, and bring coherence and alignment to their activities. Recently, the GAVI Alliance announced that it will increase its funding for strengthening health systems to US$800 million [29]. The WHO dedicated its State of the World Health Report 2008 to a renewed focus on the commitments made in Alma Ata to provide universal primary health care for all [30].

Multiple donor countries have embarked upon new initiatives to make aid more effective. The Norwegian government has recently created the Global Campaign for the Health MDGs and committed to funding one billion dollars over the next ten years towards meeting the goals of reducing child mortality, improving maternal health, and combating HIV/AIDS, malaria and other infectious diseases [31]. In September 2007, a consortium of wealthy governments and private donors announced the creation of the International Health Partnership (IHP) [32]. The IHP seeks to redesign the relationship between donors and recipient nations, to improve transparency, accountability, and cooperation in the programs executed by typically rival agencies. If the IHP succeeds, country governments will have more control over what foreign entities do within their borders, and, in return, will commit to improving all aspects of strategic planning, civil society engagement, and financial processing. The IHP promises longer term financial commitments – up to a full decade – in exchange for commitments from recipients to accountability for every dollar spent at the country level. The goal is respond more efficiently to the immediate needs of developing countries, including health infrastructures, clean water and sanitation systems, health human resources training and support, and microfinance schemes that set realistic long term goals for individual and community development [33].

As a global health community, we must stand back and objectively evaluate how we can most effectively respond to the crises of 2008 and take advantage of this moment of extraordinary attention for global health and translate it into long term, sustainable health improvements for all. On the donor side, existing commitments to global health must be upheld despite economic uncertainty. As President Bush described at a recent White House Summit, "During times of economic crisis, some may be tempted to turn inward – focusing on our problems here at home, while ignoring our interests around the world. This would be a serious mistake" [34].

Times of economic crisis necessitate a strategic evaluation of how to make each dollar, yen, or euro spent on health and development initiatives more efficient and sustainable. In an effort to make funding more impactful, donors should not put health programs – whether vertical, horizontal or diagonal – in competition with one another. For recipient countries the greatest challenges are in management: juggling precious human resources, external funds and programs, rural versus urban needs, and donor demands. The management balancing act is hard enough on a day to day basis, but must expand to encompass health infrastructure and private sector growth that function on decades long timetables. Achieving such long range strategic targets will require sustained commitment from national leaders, donors, NGOs, and private philanthropies, especially in difficult economic times.

Three decades ago, a previous momentum to put health in the forefront of the development agenda and provide access to health for all was followed by a series of economic disasters – soaring oil prices, debt crisis, multiple economic depressions, and stagflation. The international response to these crises was to enact a series of economic relief strategies that pushed developing countries further into debt and shifted their budgets away from social spending for health, education, welfare, and local infrastructures. The world became distracted from the goal of providing access to health for all, and entrenched in finding emergency, stopgap solutions, instead of tackling the larger structural determinants of poverty and disease.

In this time of financial catastrophe, the onus sits squarely on the shoulders of global health advocates living in the wealthy nations: push your governments and philanthropic institutions to not only maintain their technical and financial commitments to the poor nations of the world, but actually increase the scale of investment to reflect the rising costs of doing good in a troubled world. It is conceivable that 2008 will mark the beginning of the end of the Era of Generosity. But it is equally probable that the economic crisis will usher in a bold new era of investment in the public goods of poor and emerging market nations worldwide. Successful navigation of these turbulent waters will require a shift from the morality of "charity," to that of "change." With "charity" comes dependency and, frankly, a demeaning imbalance of power. If global health advocates seize this moment to move all priorities towards lasting change, and sustainable improvements in life expectancy and human survival, the Era of Generosity could well morph into the progressive turning point, when peoples long locked into desperate poverty and disease started on the road towards permanent transformation.

## Competing interests

The authors declare that they have no competing interests.

## Authors' contributions

Both authors (KS and LG) wrote and revised the manuscript. Both authors read and approved the final manuscript.

## Author's information

Kammerle Schneider is currently the Assistant Director of the Global Health Program at the Council on Foreign Relations in New York. Previously she had been a Refugee/IDP Program Officer for the USAID-funded Extending Service Delivery Project where she managed the agency's activities to extend reproductive health and family planning services to refugees and displaced persons. There she also served as the liaison between her agency, USAID, and the International Centre for Migration and Health in Geneva. She served as a Peace Corps volunteer in Guatemala from 2001 until 2003. Schneider holds a Master of International Affairs with a concentration in Health and Development from Columbia University, and a B.A. from the University of Washington.

Laurie Garrett is currently the Senior Fellow for Global Health at the Council on Foreign Relations in New York. Garrett is the only writer ever to have been awarded all three of the Big "Ps" of journalism: The Peabody, The Polk and The Pulitzer. Garrett is also the best-selling author of *The Coming Plague: Newly Emerging Diseases in a World Out of Balance *and *Betrayal of Trust: The Collapse of Global Public Health*. During her time as Senior Fellow for Global Health at the Council on Foreign Relations, Garrett has written several reports and articles including: HIV and National Security: Where are the Links?, A Council Report (Council on Foreign Relations Press, 2005); 'The Next Pandemic?' (*Foreign Affairs*, July/August 2005); 'The Lessons of HIV/AIDS' (*Foreign Affairs*, July/August 2005); and 'The Challenge of Global Health' (*Foreign Affairs*, January/February 2007). Garrett is a member of the National Association of Science Writers (USA), and served as the organization's President during the mid-1990s. She currently serves on the advisory board for the Noguchi Prize, François-Xavier Bagnoud (FXB) Center for Health and Human Rights (Harvard), and the Health Worker Global Policy Advisory Group. She is an expert on global health with a particular focus on newly emerging and re-emerging diseases; public health and their effects on foreign policy and national security.
